# Supplementary material for: A Free Amino Acid Diet Alleviates Colorectal Tumorigenesis through Modulating Gut Microbiota and Metabolites
Source: Nutrients. 2024 Apr 3;16(7):1040. doi: 10.3390/nu16071040 (PMC11013359; doi:10.3390/nu16071040)
Supplement: Supplementary file 1 [file nutrients-16-01040-s001.zip › nutrients-2833313-supplementary.pdf]

Table S1 The diet composition

	L-AA Defined AIN-93G		AIN-93G		
	gm%	kcal%		gm%	kcal%
Protein	18.00%	18.20%	Protein	20.30%	20.30%
Carbohydrate	65.50%	66.00%	Carbohydrate	63.95%	63.95%
Fat	7.00%	15.90%	Fat	7.00%	15.75%
Total		100.00%	Total		100.00%
kcal/gm	3.97		kcal/gm	4.00	
Ingredient	gm	kcal	Ingredient	gm	kcal
L- Arginine	6.3	25.2	Casein	200	800
L-Histidine	4.5	18	L-Cystine	3	12
L-Lysine HCl	16.1	64.4			
L-Tyrosine	9.2	36.8			
L-Tryptophan	2.1	8.4			
L-Phenylalanine	8.7	34.8			
L-Methionine	4.5	18			
L-Cystine	3.7	14.8			
L-Threonine	6.6	26.4			
L-Leucine	15.3	61.2			
L-lsoleucine	8.4	33.6			
L-Valine	9.9	39.6			
Glycine	3.1	12.4			
L-Proline	20.4	81.6			
L-Glutamic Acid	36.2	144.8			
L-Alanine	4.5	18			
L-Aspartic Acid	11.3	45.2			
L-Serine	9.4	37.6			
Total L-Amino Acid	180.2	720.8			
Corn starch	399.9	1599.6	Corn starch	397.5	1590
Maltodextrin	145	580	Maltodextrin	132	528
Sucrose	100	400	Sucrose	100	400
Cellulose	50	0	Cellulose	50	0
Soybean Oil	70	630	Soybean Oil	70	630
Choline Bitartrate	2.5	0	Choline Bitartrate	2.5	0

Mineral Mix S10022G	35	0	Mineral Mix S10022G	35	0
Vitamin Mix V10037	10	40	Vitamin Mix V10037	10	40
Sodium Bicarbonate	7.4	0			
Total	1000	3970.4	Total	1000	4000

Table S2 Differential Gene Expression Analysis

Gene ID	Gene Symbol	Log2FC	Qvalue	Expression
100041596	Gal3st2b	1.714988801	0.0488955	Up
100061	Lrrc19	1.10166328	9.48E-04	Up
100763	Ube3c	0.46134953	4.06E-04	Up
100986	Akap9	0.567304175	0.008332666	Up
102633156	Gm4631	3.85380332	0.014566529	Up
102680	Slc6a20a	1.703151272	5.33E-04	Up
104263	Kdm3a	0.434531237	0.022999129	Up
105244158	1700024J04Rik	1.924811915	1.95E-04	Up
105244931	Gm40453	2.572830359	8.95E-04	Up
105440	Kctd9	0.440858521	0.00302005	Up
106583	Scaf8	0.359348581	0.003905969	Up
107035	Fbxo38	0.395985056	4.88E-07	Up
107371	Exoc6	0.537907213	4.69E-04	Up
107895	Mgat5	0.623452183	7.04E-04	Up
108017	Fxyd4	4.730397294	0.046272555	Up
108654	Fam210a	0.32677108	0.013068687	Up
109263	Rlf	0.701179133	0.011103541	Up
109333	Pkn2	0.426238346	0.027424311	Up
112407	Egln3	1.156817459	0.001283779	Up
11306	Abcb7	0.414987392	0.04509765	Up
115488029	LOC115488029	1.763770864	0.018706587	Up
11765	Ap1g1	0.381828954	0.004377666	Up
11798	Xiap	0.47463572	0.044931394	Up
118567641	LOC118567641	0.899793292	0.022779793	Up
118568652	LOC118568652	1.715646342	1.22E-06	Up
118568653	LOC118568653	1.368516049	0.042937529	Up
12013	Bach1	0.673259287	0.006210752	Up
12211	Birc6	0.310290669	0.038660178	Up
12695	Patj	0.303632111	0.030124146	Up
13383	Dlg1	0.263658222	0.007861662	Up
13521	Slc26a2	1.319619234	0.028724885	Up
13709	Elf1	0.330925471	0.028724885	Up
13714	Elk4	0.95020811	0.031182561	Up
14107	Fat1	0.41005718	0.039148423	Up
14479	Usp15	0.321317376	0.006501409	Up
14544	Gda	1.341614041	4.88E-07	Up
14675	Gna14	1.154706968	0.00302005	Up
14933	Gk	1.790274277	3.26E-04	Up
15490	Hsd17b7	0.797605275	0.035296468	Up
16364	Irf4	1.357661918	0.002859531	Up

16396	Itch	0.41033752	0.025989125	Up
16589	Uhmk1	0.401387034	0.005002044	Up
16601	Klf9	0.733221812	0.028724885	Up
16643	Klrd1	1.348419201	0.042937529	Up
16780	Lamb3	0.291235842	4.69E-04	Up
17126	Smad2	0.430370514	0.001863304	Up
17128	Smad4	0.665022241	0.0167029	Up
17295	Met	0.775243298	0.026800914	Up
17356	Afdn	0.347576229	0.014566529	Up
17847	Usp34	0.472479812	0.023075992	Up
17909	Myo10	0.296604018	0.001863304	Up
17948	Naip2	0.534427272	0.009394644	Up
18021	Nfatc3	0.313029733	0.039552133	Up
18174	Slc11a2	1.242495984	0.045059137	Up
18585	Pde9a	0.797813122	0.0167029	Up
18607	Pdpk1	0.41998834	0.00302005	Up
18700	Piga	0.500915026	0.0488955	Up
18708	Pik3r1	0.452479588	0.011392109	Up
19087	Prkar2a	0.52412618	5.33E-04	Up
19260	Ptpn22	1.53062026	0.00781177	Up
19271	Ptprj	0.579452574	0.022779793	Up
19272	Ptprk	0.35579751	0.043440155	Up
192786	Rapgef6	0.411050859	0.041427534	Up
19386	Ranbp2	0.465552042	0.035936508	Up
19766	Ripk1	0.289029544	0.001711529	Up
20278	Scnn1g	4.452967713	2.04E-09	Up
20338	Sel1l	0.328286207	0.040248805	Up
20384	Srsf5	0.300200406	0.022961417	Up
20393	Sgk1	1.474897162	0.022779793	Up
20658	Son	0.366879457	0.042841322	Up
20683	Sp1	0.368148391	0.00375101	Up
20717	Serpina3m	3.983814826	0.004359487	Up
207214	Larp4	0.540628924	0.026800914	Up
207304	Hectd1	0.424158208	0.010908776	Up
208618	Etl4	0.410036586	0.047249558	Up
211673	Arfgef1	0.375801312	0.02332722	Up
212285	Arap2	0.774179488	0.024660227	Up
212391	Lcor	0.3369195	0.031182561	Up
212442	Lactb2	0.40994715	0.011911087	Up
212728	Tarbp1	0.736454855	0.013541765	Up
215335	Slc36a1	1.240204113	0.012179448	Up
216238	Eea1	0.656542702	0.035936508	Up
217030	Synrg	0.313092001	0.009371183	Up

217980	Larp4b	0.44195957	7.01E-04	Up
21872	Tjp1	0.501075152	0.005785178	Up
21888	Tle4	0.96240124	4.40E-05	Up
218914	Wapl	0.383940986	0.035936508	Up
22026	Nr2c2	0.742482365	6.59E-06	Up
223770	Brd1	0.196166729	0.031182561	Up
225742	St8sia5	4.780932791	0.025989125	Up
226641	Atf6	0.48778741	0.007589987	Up
227231	Cps1	3.092078747	5.04E-04	Up
227638	Qsox2	0.38549133	0.010944415	Up
228850	Ralgapb	0.287726221	0.024816344	Up
229731	Slc25a24	0.510215667	0.026536814	Up
231876	Lmtk2	0.364255581	0.025548324	Up
233789	Smg1	0.563447387	0.043019643	Up
235320	Zbtb16	1.786060046	4.35E-05	Up
235567	Dnajc13	0.346438669	0.003921589	Up
235626	Setd2	0.318560338	0.010908776	Up
238871	Pde4d	0.52424924	0.025922347	Up
240255	Ythdc2	0.601960287	0.031182561	Up
240283	Dmxl1	0.470697899	0.013541765	Up
240328	F830016B08Rik	0.883788621	0.033328904	Up
242291	Bpnt2	0.516366458	0.007225062	Up
243983	Zdhhc13	0.756047101	0.00781177	Up
244349	Kat6a	0.409984596	0.015350992	Up
244373	Erlin2	0.280732458	0.049495328	Up
246103	Atxn7	0.368249681	0.035296468	Up
268420	Alkbh5	0.294654953	0.030124146	Up
268656	Sptlc1	0.33578635	0.011392109	Up
268973	Nlrc4	0.496946254	0.042937529	Up
268980	Strn	0.402895263	0.024785686	Up
269023	Zfp608	0.573974145	0.00302005	Up
27392	Pign	0.546019869	0.038505872	Up
29815	Bcar3	0.539033969	0.00302005	Up
30962	Slc7a9	1.791157219	0.001420454	Up
317652	Klk15	5.537924825	0.009560463	Up
319448	Fndc3a	0.645239086	0.010823196	Up
320506	Lmbrd2	0.64416625	0.010233818	Up
320615	Dop1a	0.577483737	1.94E-08	Up
320727	Ipo8	0.339107144	0.009700473	Up
329002	Zfp236	0.389857408	7.50E-04	Up
330914	Arhgap32	0.60773808	0.03945778	Up
332131	Krt78	1.455832066	0.040650199	Up
381213	Ms4a12	1.639128325	0.009951678	Up

381246	Xkr9	1.075411956	0.005002044	Up
381334	Gal3st2	1.968387628	2.79E-04	Up
50527	Ero1a	0.773088618	0.013541765	Up
50877	Neu3	2.151621617	0.034839792	Up
52036	Ppp6r3	0.283996049	0.015642638	Up
52440	Tax1bp1	0.631746624	0.002371804	Up
53417	Hif3a	1.423808321	0.001711529	Up
53608	Map3k6	0.796698719	0.003883798	Up
53945	Slc40a1	1.147055587	0.006501409	Up
544696	Tbc1d32	0.504578672	0.039140712	Up
54608	Abhd2	0.325636323	0.027031249	Up
54670	Atp8b1	0.327427968	0.026579543	Up
56406	Ncoa6	0.43134565	2.27E-06	Up
56542	Cilk1	0.481567152	0.021465873	Up
56637	Gsk3b	0.454178122	0.023355869	Up
56736	Rnf14	0.386188969	0.016237965	Up
60599	Trp53inp1	0.817771981	0.021595244	Up
619597	Gal3st2c	2.413604722	0.033880959	Up
66222	Serpinb1a	0.665435801	0.042937529	Up
66595	Aste1	0.445727725	0.012111701	Up
66691	Gapvd1	0.360841191	0.045059137	Up
667118	Zbed6	1.449567453	0.001352913	Up
67204	Eif2s2	0.850648282	0.013068687	Up
67300	Cltc	0.507019227	0.012163609	Up
67974	Ccny	0.344597621	0.00302005	Up
68142	Ino80	0.419710937	0.025251425	Up
68473	Mob1b	1.023456976	3.09E-04	Up
68795	Ubr3	0.356832965	0.019244457	Up
68813	Dock5	0.60885936	0.042937529	Up
69562	Cdk13	0.345911508	0.025464035	Up
70349	Copb1	0.360416027	0.00302005	Up
70351	Ppp4r1	0.390477468	0.00302005	Up
70829	Ccdc93	0.751038662	0.042841322	Up
71756	Cpn2	3.71036958	0.016050948	Up
71844	Nupl1	0.549731625	0.007870617	Up
71903	Ces2f	2.651972691	0.010908776	Up
72503	2610507B11Rik	0.333413527	0.042937529	Up
73288	Vps50	0.398065947	0.026536814	Up
74006	Dnm1l	0.386699871	0.043440155	Up
74132	Rnf6	0.244397889	0.0208358	Up
74143	Opa1	0.420253693	7.17E-06	Up
74388	Dpp8	0.290006861	0.047249558	Up
74737	Pcf11	0.377900276	0.018726474	Up

75420	Secisbp2	0.341014024	0.043019643	Up
75805	Nln	0.430378425	0.010908776	Up
76295	Atp11b	0.442421998	0.043019643	Up
76740	Efr3a	0.378975129	0.030075334	Up
76781	Mettl4	0.514306296	0.040773257	Up
76983	Scfd1	0.422894962	0.001570418	Up
77963	Hook1	0.586943596	0.042937529	Up
78937	Avl9	0.791779165	0.008936583	Up
80877	Lrba	0.466483581	0.037055423	Up
80898	Erap1	0.536117219	0.019492742	Up
81898	Sf3b1	0.280058452	0.025663314	Up
83921	Cemip2	0.445056714	0.013068687	Up
94192	C1galt1	0.885262246	0.002338826	Up
94212	Pag1	1.181361969	0.001377421	Up
99929	Tiparp	0.731305818	0.002444147	Up
100689	Spon2	-1.248127885	3.46E-08	Down
101543	Wtip	-0.673960481	0.013541765	Down
107765	Ankrd1	-2.226445078	0.039078992	Down
109959	Amy2a5	-3.486539732	0.036294033	Down
110962	Mbd6	-0.274676112	0.027424311	Down
116847	Prelp	-0.71351354	0.031182561	Down
12009	Cep131	-0.69160636	1.95E-04	Down
12798	Cnn2	-0.673652803	0.003214312	Down
13542	Dvl1	-0.312445033	0.004185172	Down
13543	Dvl2	-0.489492371	0.023706135	Down
13644	Efs	-0.926554022	0.020038706	Down
13706	Cela2a	-2.72677172	0.037055423	Down
13852	Stx2	-0.774950085	3.09E-04	Down
14114	Fbln1	-0.493118837	0.031182561	Down
14371	Fzd9	-1.797164902	0.006572642	Down
14573	Gdnf	-1.786066322	0.040248805	Down
14581	Gfi1	-0.701748018	0.015354202	Down
14588	Gfra4	-1.488655027	0.019492742	Down
15248	Hic1	-0.641700446	4.78E-05	Down
15370	Nr4a1	-0.964188317	0.024553866	Down
15396	Hoxa11	-0.642163856	1.50E-04	Down
15402	Hoxa5	-0.699676	0.034180445	Down
15404	Hoxa7	-0.474909905	0.031182561	Down
16542	Kdr	-0.450491603	0.0313254	Down
16779	Lamb2	-0.730306456	0.033706551	Down
16949	Loxl1	-0.842655045	0.029013734	Down
16950	Loxl3	-0.744047218	0.006572642	Down
16998	Ltbp3	-0.761464783	0.049572743	Down

170770	Bbc3	-0.633208311	0.012931574	Down
17283	Men1	-0.448613839	0.036530773	Down
17769	Mthfr	-0.652670631	1.41E-05	Down
17937	Nab2	-0.779850359	0.023075992	Down
18029	Nfic	-0.527924442	0.041659553	Down
18146	Npdc1	-0.419445211	0.041427534	Down
18515	Pbx2	-0.33183262	0.026579543	Down
18709	Pik3r2	-0.341580597	0.001711529	Down
192654	Pla2g15	-0.717532271	0.029553315	Down
19335	Rab23	-0.830442686	0.042937529	Down
19731	Rgl1	-0.626591264	0.002892641	Down
19732	Rgl2	-0.428961904	0.013685383	Down
20289	Scx	-1.515019712	0.015379441	Down
20364	Selenow	-0.672118585	0.009821046	Down
20441	St3gal3	-0.998000066	0.008763022	Down
20472	Six2	-3.083303469	0.013541765	Down
20475	Six5	-0.902808834	0.037817558	Down
20667	Sox12	-0.91128609	0.018207929	Down
20672	Sox18	-1.146656552	0.01468612	Down
207259	Zbtb7c	-0.573788841	0.001801206	Down
207792	BC034090	-0.930294052	0.024154909	Down
21412	Tcf21	-1.135256581	3.09E-05	Down
217333	Trim47	-0.844512733	0.018706587	Down
21809	Tgfb3	-1.082171943	0.040002919	Down
22021	Tpst1	-0.607421885	0.0488955	Down
22074	Try4	-5.01032195	0.028724885	Down
22368	Trpv2	-0.845670501	0.042773666	Down
223690	Ankrd54	-0.366823727	0.033328904	Down
224024	Scarf2	-0.821938199	0.007870617	Down
224116	Muc20	-0.688649814	0.024785686	Down
224697	Adamts10	-0.590449124	0.041578633	Down
226154	Lzts2	-0.460704235	0.002084455	Down
22750	Zfp9	-0.776917908	0.027698339	Down
229949	Ak5	-1.58030135	0.031182561	Down
230837	Asap3	-0.668385453	0.021595244	Down
232664	Ccdc136	-1.165644316	0.027031249	Down
234395	Ushbp1	-0.682274142	0.002338826	Down
234582	Ccdc102a	-0.890064153	0.018726474	Down
23876	Fbln5	-0.662949221	0.017923079	Down
240057	Syngap1	-0.785150455	0.034180445	Down
240888	Gpr161	-0.998651556	0.00302005	Down
244654	Mtss2	-1.280172179	0.042937529	Down
269881	Map3k10	-0.501141349	0.035936508	Down

27801	Zdhhc8	-0.72808775	2.57E-04	Down
319655	Podxl2	-1.353856665	0.013541765	Down
320078	Olfml2b	-1.078342103	0.021146184	Down
330790	Hapln4	-2.334470991	0.02753401	Down
442801	Arhgef15	-0.841814211	1.50E-04	Down
52163	Camk1	-0.379182229	0.029981704	Down
53412	Ppp1r3c	-1.124406864	0.033748918	Down
54201	Zfp316	-0.513341275	0.023509394	Down
56325	Abcb9	-0.785171383	0.040248805	Down
56327	Arl2	-0.617990482	0.019244457	Down
56350	Arl3	-0.659514157	0.021465873	Down
56437	Rrad	-1.611755864	2.98E-04	Down
56516	Rbms2	-0.373052443	0.00909586	Down
57265	Fzd2	-1.200776414	0.002377709	Down
59016	Thap11	-0.462947418	0.017923079	Down
59047	Pnkp	-0.331839634	0.001673839	Down
59092	Pcbp4	-0.695449064	0.013068687	Down
613123	Ugt1a8	-24.84142592	4.13E-12	Down
619441	Tnfsfm13	-0.993428988	0.024154909	Down
65086	Lpar3	-1.536081443	0.032416191	Down
65962	Slc9a3r2	-0.777220946	0.049167639	Down
66220	Zdhhc12	-0.479564018	0.048490584	Down
66473	Ctrb1	-4.427980191	0.006501409	Down
66775	Hacd4	-0.948197597	0.025706414	Down
668303	Kif26a	-1.487335595	0.009701624	Down
66873	Tril	-0.819734887	0.031182561	Down
67373	2210010C04Rik	-5.106666059	0.048008899	Down
67622	Mxra7	-0.772796164	0.044378999	Down
68106	Nt5c3b	-0.446652055	0.00595821	Down
68337	Crip2	-0.645711834	0.032416191	Down
68427	Slc39a13	-0.615094274	0.006775006	Down
68490	Zfp579	-0.445228295	0.042937529	Down
68588	Cthrc1	-2.381570772	0.013676411	Down
68632	Myct1	-1.232137733	0.001711529	Down
68767	Washc1	-0.376404777	0.01252806	Down
68794	Flnc	-1.826374275	0.029900178	Down
68977	Haghl	-0.524285783	0.018794362	Down
69060	Pnlip	-4.276662668	0.013541765	Down
69202	Ptms	-0.549808725	9.89E-08	Down
71709	Syde1	-0.686066158	0.049572743	Down
72000	Lmntd2	-0.449981062	0.043019643	Down
72446	Prr5l	-1.20200592	0.025548324	Down
73181	Nfatc4	-0.946183879	0.010973094	Down

73822	Mfsd12	-0.806476527	0.022779793	Down
74270	Usp20	-0.439438552	0.01199322	Down
74463	Exoc3l2	-1.333487839	1.95E-04	Down
75687	Ripor1	-0.491250165	0.042841322	Down
75871	Zfp821	-0.567459269	0.012131447	Down
78070	Cpt1c	-0.79558954	0.029981704	Down
78248	Armcx1	-0.637713949	0.029553315	Down
78408	Fam131a	-1.11498017	0.029029981	Down
81799	C1qtnf3	-0.803059576	0.037824652	Down
83396	Glis2	-0.92594039	1.78E-04	Down
84113	Ptov1	-0.455265259	0.00781177	Down
97440	B3gnt9	-0.816991316	0.03516691	Down
99151	Cercam	-1.235249859	0.016644199	Down

Table S3 Forward and reverse primer sequences for EGLN 3

**Forward Primer**	CTGGGCAAATACTACGTCAAGG
**Reverse Primer**	GACCATCACCGTTGGGGTT
